# Polychlorinated biphenyls (PCBs) residues in commercial pasteurized cows’ milk in Tehran, Iran

**DOI:** 10.1186/s40201-017-0278-y

**Published:** 2017-07-04

**Authors:** Reza Ahmadkhaniha, Ramin Nabizadeh Nodehi, Noushin Rastkari, Hassan Mohammadi Aghamirloo

**Affiliations:** 1 Department of Human Ecology, School of Public Health, Tehran University of Medical Sciences, Tehran, Iran; 2 Environmental Health Department, School of Public Health, Tehran University of Medical Sciences, Tehran, Iran; 3 Center for Air Pollution Research (CAPR), Institute for Environmental Research (IER), Tehran University of Medical Sciences, Tehran, 1417993359 Iran

**Keywords:** Polychlorinated biphenyls, Milk, Daily intake

## Abstract

**Background:**

To date, despite the facts that pasteurized milk is the most consumed dairy product in Iran and its consumption has increased almost two fold during the last 10 years, no data are available concerning the concentrations of polychlorinated biphenyls (PCBs) in commercial cow milk in Iran market.

**Methods:**

This study designed to determine the levels of PCBs in these products and to assess population exposure to PCBs by estimating the daily intakes. Pasteurized cows’ milk samples (10 brands) were collected from local markets at two different seasons and analyzed using sensitive and reliable methods.

**Results:**

Based on the results all the indicator PCBs were detected and quantified in all of the samples, the mean ± SD concentration for the sum of the six congeners was 18.92 ± 14.36 ng g^−1^ fat. None of the samples surpassed the provisional value established by the EU of 40 ng g^−1^ fat. The sum of dioxin-like congeners, expressed as WHO-TEQ was 0.492 pg/g of fat which was considerably lower than the defined limit 3 pg/g fat, set for cow’s milk. Furthermore, a similar DL-PCBs profile as other studies was found for analyzed samples. The results indicated that concentrations of DL-PCBs were very low, and all of milk samples were compliant with EC legislation. In addition, seasonal variations were not observed for DL- and NDL-PCBs levels (*p* values >0.05).

**Conclusions:**

The estimated dietary intake for target population was 0.06 pg TEQ/kg of body weight/day, much smaller than the amounts declared by the World Health Organization as tolerable daily intake.

## Background

Polychlorinated biphenyls (PCB) make up a highly toxic group of pollutants that has widely spread in the environment. Many of human activities are the main causes of this kind of pollution and many evidences about adverse biological and toxic effects of PCBs have been found. PCBs may cause cancer development, immune deficiency disorders and reproductive, nervous or other biological systems malfunction [1, 2]. PCBs consists a group of several different congeners, which are divided into two main groups on the basis of their chemical and toxicological properties. A number of 12 PCB congeners (including PCB77, PCB81, PCB 126, PCB169, PCB105, PCB114, PCB118, PCB123, PCB156, PCB157, PCB167 and PCB189) show toxicological effects similar to dioxins and therefore are named “dioxin-like PCBs” (DL-PCBs). The other group of PCBs which is comprised of six congeners (including PCB28, PCB52, PCB101, PCB138, PCB153 and PCB180) named “indicator PCBs” and usually are selected as suitable markers for PCBs pollution studies [3]. Indicator PCBs are predominantly present in biotic and abiotic matrices and therefore are usually used as pollution indicator for surveys [4]. PCBs can be released into the environment and finally enter the food chain by means of the manufacturing and industrialized processes, daily usage of synthetic products, waste disposal and other human activities [5, 6]. Previous studies have shown that food is a major route of human exposure to these compounds which are lipophilic and have the propensity to bio-accumulate in biota [7–9]. Generally, fatty foods of animal origin are the major sources of human exposure to lipophilic contaminants [10, 11]. Grazing cows ingest contaminated grass, hay and silage then the contaminants are absorbed from the gastrointestinal tract and distributed into the fat compartments of cow’s body including the milk fat of the lactating cow [12]. Milk fat is likely to be one of the major foodstuffs that mainly contribute to the human exposure to PCBs, thus, consumption of milk and its products could significantly contribute to the dietary intake of PCBs. In the case of children, this rout of exposure as well as the extent of harmful effects could be significant [12–14]. European Union (EU) regulation no. 1259/2011 on food safety established maximum levels for a number of contaminants in foodstuffs including PCBs. The values were expressed in term of toxic equivalents (TEQ) which are based on WHO-TEF (WHO-toxic equivalency factors) established in 2005 [15]. Based on the regulation, milk and dairy products must not contain dioxin-like PCBs levels higher than 5.5 pg TEQ/g fat. In addition the provisional maximum levels of 40 ng/g of milk fat has been established for the sum of the indicator PCBs or non-dioxin-like PCBs (NDL-PCBs). Considering that up to now, no study has been conducted in Iran for determining PCBs levels in commercial pasteurized milk, the aim of this research is to determine the levels of PCBs in commercial cow milk consumed in Tehran and to assess population exposure to PCBs by estimating the toxic equivalents (TEQ).

## Methods

### Preparation of milk and sampling

One hundred twenty Pasteurized cows’ milk samples from 10 brands (full fat grade, contained around 3% fat) purchased from high delivery rate supermarket of Tehran, Iran, at two different seasons (spring and fall, 60 sample in each season) 2015. Each of the ten selected brands was sampled several times during sampling time in order to obtain a representative estimation for each brand and to study potential variations among different batches. Packaging material of samples was either tetra pack or polyethylene type. All the samples were stored in fridge at 4 °C not more than 2 days until analysis. Sample containers (non-pooled) were gently shaken before taking aliquot for analysis and backup sample. Total lipid of each sample was determined on a separate aliquot of the sample according to the AOAC method [16]. Briefly 1.5 mL NH_4_(OH) was mixed with 10 grams of milk. Three drops of phenolphthalein indicator and 10 mL of 95% alcohol were added and shaken. Then, 25 mL diethyl ether was added and shaken vigorously for 1 min, afterward; 25 mL petroleum ether was added and shaken. The mixture was centrifuged at 600 rpm for 30 s and the upper layer was collected. For the second extraction, again, 5 mL of 95% alcohol was added and shaken for 15 s; diethyl ether (15 mL) was added, shaken, and centrifuged at 600 rpm for 30 s. For the third extraction, the same procedure was followed as in second extraction.

### Reagents

A standard DL- PCBs mixture solved in nonane (IUPAC 77, 81, 105, 114, 118, 123, 126, 156, 157, 167, 169, 180 and 189) and standards of five indicator PCBs (IUPAC 28, 52, 101, 138 and 153) were obtained from AccuStandard (New Haven, CT). SPME fibers coated with 100 μm (PDMS) were obtained from Supelco (Bellefonte, PA, USA). SPME fibers were conditioned prior to use according the supplier’s instructions. To construct the calibration lines, some organic milk samples were first analyzed to determine any background levels then were spiked with known amounts of the PCBs. The samples were cooled at about 4 °C for 24 h (to enable the active biological milk compounds to bind to the added PCBs) before being analyzed, as described in the previous work [17]. For the optimization studies, the spiked concentration was 10 μg L^−1^; for the validation experiments, blank samples were spiked with amounts ranging from 1 to 20 μg L^−1^ of the analytes. Hexachlorobenzene was used as internal standard (I.S.) and prepared in methanol (5 μg L^−1^).

### Instrumental analysis

Target PCBs were determined using gas chromatograph (Agilent 6890 Series, Agilent, Palo Alto, CA) equipped with micro electron capture detector. Separation was performed on a DB-5 capillary column (60 m length, 0.25 mm i.d., 0.25 μm film thickness, J&W Scientific, USA). The initial oven temperatures was set at 80 °C for 2 min then increased to 185 °C at a rate of 30 °C min^−1^ (3 min), then at 1.5 °C min^−1^ to 230 °C (held for 15 min) and finally to 270 °C at a rate of 5 °C min^−1^ (held for 25 min). Nitrogen as carrier gas was used at 1.8 mL min^−1^ flow rate. Injector was set at 270 °C and split-less mode. Detector was set at 300 °C. Confirmation of target PCB congeners were carried out using a Varian Gas Chromatograph CP-3800 (Varian, CA, USA) coupled to ion trap mass spectrometry detector (Varian Saturn 2000, Varian, CA, USA). The mass spectrums were acquired in MS/MS mode. 2 μL of each sample was injected in a programmable temperature vaporizing (PTV) injector (split-less mode). A VF-5MS capillary column (55 m length, 0.25 mm i.d. and 0.25 μm film thicknesses, Factor Four ®, Varian, Palo Alto, CA, USA) was employed. The GC oven was programmed as: initial temperature 100 °C for 2 min then to 200 °C (held for 3 ° min) at a rate of 30 °C min^−1^ and then to 230 °C (held for 15 min) at a rate of 3 °C min^−1^ and finally to 270 °C (held for 15 min) at a 5 °C min^−1^ [18, 19].

### SPME procedure

To 5 mL of sample in a headspace vial (20 mL), methanol (5.0%, v/v) and NaCl (36%, w/v) were added. After magnetic stirring of the sample for 10 min at 1200 rpm, the PDMS-SPME fiber was exposed to the headspace for 60 min at 96 °C. After adsorption phase the fiber was withdrawn and the analytes were immediately desorbed directly in the GC injection port at 290 °C. Based on the results of optimization study, desorption step will end at 1 min. however, to prevent carryover effect; the fibers were kept in the injector for extra 3 min before next sample run. The suitability of this procedure was confirmed by performing blank runs before each sample run.

### Statistical analyses

A simple statistical analysis was carried out for the mean, standard deviation, minimum and maximum values using SPSS statistical packages. The non-parametric Kruskal-Wallis test was carried out to compare the PCBs concentrations between sampling season. The level of significance was set to 0.05 and *p* >0.05 were assumed to be not statistically significant. The toxic equivalent (TEQ) for the dioxin analogues in the analyzed samples were calculated using the World Health Organization (WHO)-2005 toxic equivalency factors (TEF) [20]. The concentration of the congeners below the LOQ was calculated using the lower and upper bound approach, which means that the values below LOD/LOQ will be replaced by zero for the lower bound and by the LOD/LOQ for the upper bound [21]. The toxic equivalency of a mixture is defined by the sum of the concentrations of individual compounds (Ci) multiplied by their relative toxicity (TEF) [22, 23].

1 $$ \mathrm{T}\mathrm{E}\mathrm{Q} = \Sigma\ \left[\mathrm{Ci}\right] \times \mathrm{TE}\mathrm{Fi} $$

## Results

**Fig. 1:**
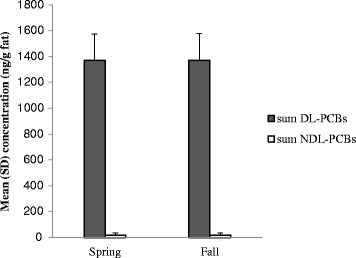
Seasonal variations in concentrations (Mean ± SD) of PCBs in milk samples

Determination of the total TEQ were carried out based on assumption that all non-detected (n.d.), as well as smaller than limit of quantification values (<LOQ), were absent (lower bound value). A congener was considered as “non-detected” when the signal to noise ratio (S/N) for its given peak was lower than 3. The limits of detection (LODs) were evaluated using either average method blank values or values of smaller added concentration giving a signal with signal to noise ratio greater than 3 when average method blank values were too low [13]. LODs were defined as this S/N > 3 value plus 3 times the standard deviation (SD) of the blank. LOQs were defined as this S/N > 3 value plus 10 times the SD of the blank. When the difference between lower and upper bound was not higher than 10% the LOQ values were taken into account to estimate the contribution of each n.d. congeners (upper bound approach). All the NDL-PCBs including: PCB28, PCB52, PCB 101, PCB 153, PCB 138 and PCB 180 were detected and quantified in the samples which indicate that each congener concentration and their total amount represent suitable markers for exposure studies. The mean NDL-PCBs (indictor PCBs) level, based on the sum of concentration of six congeners in all samples (*n* = 120) was 1.8 ± 1.4 ng g^−1^ fat (Table 1). Mean concentrations and WHO-TEQ levels of target DL-PCBs measured in collected samples are presented in Table 2. Evaluation of toxicity of DL-PCBs congeners were carried out according to the definition of the WHO-TEF [20]. Based on the results significant differences (*p* values >0.05) were not observed in terms of seasonal variation for NDL-PCB levels (Fig. 1).

**Table 1:** Results of the analytical determinations (Mean ± SD) expressed as (ng g^−1^ fat) for NDL-PCBs (*n* = 120)

NDL-PCBs	Mean	Min	25th	50th	95th	Max
PCB28	1.04 ± 0. 69	0.20	0.60	0.85	2.39	2.60
PCB52	0.87 ± 0.63	0.20	0.41	0.82	2.67	2.70
PCB101	0.96 ± 0. 64	0.25	0.38	0.91	3.27	3.60
PCB138	2.41 ± 1.37	0.20	0.56	2.01	2.99	3.05
PCB153	7.53 ± 4.57	0.30	1.17	7.04	11.02	12.17
PCB180	6.11 ± 3.62	0.20	0.92	6.10	12.91	13.28
Total NDL-PCBs	18.92 ± 14.36	1.35	4.04	17.73	35.25	37.40

**Table 2:** Results of the analytical determinations (Mean ± SD) expressed as (ng g^−1^ fat) and WHO-TEQ (TEQ pg g^−1^ fat) levels of 12 DL-PCBs (*n* = 120)

DL-PCBs	Mean	Min	25th	50th	95th	Max	WHO-TEQ levels
PCB77	3.49 ± 2.95	0.5	1.75	3.11	18.31	22.43	0.0035
PCB81	2.77 ± 2.67	0.5	1.02	2.64	17.96	20.11	0.00028
PCB126	2.58 ± 3.68	0.5	0.95	2.41	6.12	8.32	0.258
PCB169	4.34 ± 6.18	0.5	2.94	4.44	15.69	19.81	0.0434
Total non-ortho DL-PCBs	13.18 ± 11.41	2.0	6.66	12.60	58.08	70.67	0.3
PCB105	330.63 ± 45.69	0.75	59.25	250.21	741.36	811.41	0.0331
PCB114	18.96 ± 15.18	0.75	7.68	15.29	66.41	70.35	0.0095
PCB118	850.36 ± 240.49	0.5	69.12	659.38	1310.11	1360.23	0.085
PCB123	17.35 ± 5.48	0.5	4.23	15.47	26.37	28.01	0.00174
PCB156	93.05 ± 48.72	0.75	31.24	99.12	121.38	127.21	0.0465
PCB157	26.76 ± 8.00	0.75	10.32	28.45	40.12	42.15	0.0134
PCB167	15.68 ± 16.49	0.75	8.12	11.28	48.62	58.51	0.00016
PCB189	5.60 ± 8.44	0.75	2.51	4.95	12.35	42.53	0.00056
Total ortho DL-PCBs	1358.39 ± 179.86	5.50	192.47	1084.15	2366.72	2540.4	0.19
Total DL-PCBs	1371.57 ± 297.02	7.50	199.13	1096.75	2424.80	2611.07	0.49

## Discussion

The purpose of this research was to assess the situation of PCBs pollution in commercial pasteurized milk and to verify whether the concentration of PCBs in the samples comply with EU maximum allowable levels. These are critical questions should be answered before deciding which measures to take? This is the first study in Iran aiming to determine simultaneously the levels of DL-PCBs, and NDL-PCBs in commercial pasteurized cows’ milk samples.

### NDL-PCB concentrations

None of the samples exceeded the maximum level of 40 ng g^−1^ fat established by EU Regulation for the sum of the indicator NDL-PCBs in raw milk [15]. The mean of the sum of NDL-PCBs in 5640 collected milk products in different European countries was 9.2 ± 3.4 ng g ^−1^ fat [24]. At first look it seems that the levels of NDL-PCBs in Iran samples are considerably lower than the EU samples. In all the samples, the NDL-PCBs profile was characterized by the prevalence of PCB 180, followed by PCB 153 and PCB 138, while the remaining congeners (PCB 28, PCB 52 and PCB 101) were present at lower concentration. PCB 153 and PCB 180 appeared to contribute to more than 75.5% to the sum of NDL-PCB concentration similar to European dairies [25]. PCB180, the heptachlor congener, with octanol-water partition coefficient (Kow) of 6.7-7.2, has a high affinity for lipophilic material and therefore would be concentrated in high fat matrices such as cow milk [26]. PCB153, the other mostly detected compound in the samples, has a lower partition coefficient than PCB180 and consequently concentrates at lower concentration than PCB180 in the milk. These results are in accordance with the results of previous studies referring to the positive correlation between distribution of the PCBs in different matrices and their octanol-water partition coefficients [27–29]. Furthermore, the value of NDL-PCB contamination, determined in our study was lower than the values reported by other studies. For example a mean value around 4 ng/g of fat was reported for cow milk in some of European countries [30]. As well as studies conducted in northern European countries, some other similar studies concluded that, the main source of exposure to PCBs is dairy products [24]. Interestingly, the prevalence of congeners 138 and 153 in the collected samples, similar to our results, are highlighted by those studies. As shown in Fig. 1, the significant differences (*p* values >0.05) were not observed in terms of seasonal variation for NDL-PCB levels. These findings are in contrary to other studies which reported increasing level of PCBs in milk in winter [31]. An explanation for this result is the routine system for cow feeding in Iran. Due to limitation of natural pastures in Iran, usually dried hay, silage and crops are used for cow feeding instead of natural grass. Dried hay, silage and other crops are usually provided from limited sources and distributed between dairy farms. Therefore, most of the farms use the same source of crops for cow feeding at the same time and this could be the reason for narrow range of values obtained for NDL-PCBs levels in this study.

### DL-PCB concentrations

The sum of dioxin-like congeners, expressed as WHO-TEQ was 0.492 pg/g of fat. This value is considerably lower than the defined limit 3 pg/g fat, set for cow’s milk [32]. Levels of contaminants determined in this study are similar to those reported by some other studies: 0.16 pg/g of fat in Finland, 0.18 in Greece, 0.65 in Catalonia, Spain, 0.31 in Sweden and 0.43 in the United Kingdom [7, 33–35]. Higher values were reported for DL-PCBs in cow milk by some of other studies [36, 37] Assessment of DL-PCBs profile in the samples indicated that mono-ortho congeners were more abundant than the non-ortho ones. Furthermore congener 118 was the dominant chemicals (over 80%).

The highest contribution to the total value is from PCB-126 (estimated 52.5% of the sum of the 12 DL-PCBs). Among mono-ortho DL-PCBs, PCB 118 showed the highest concentration. A similar DL-PCBs profile was found in milk samples collected in Italy [38]. They also found that the mono-ortho congeners were more abundant than the non-ortho ones. The similarity of the congeners pattern may be due to similar sources of emissions such as similar industries or factories in different countries. The other reason could be using of similar source of silage and crops for cow feeding. Based on the report of ministry of industry, mine and trade of Iran a part of silage and crops for cow feeding are provided by import [39]. Based on the results the levels of DL-PCBs were very low, and all of milk samples were compliant with EC legislation. In addition seasonal variations were not observed for DL-PCB levels in tested samples (*p* values >0.05) (Fig. 1). Milk fat and dairy products contribute from 5.2 to 80% to the total daily intake of general population, depending on the eating habits [40–42]. According to the Iranian Agricultural Ministry, a monthly consumption of 8 L of milk or 240 g milk fat *per capita* can be assumed as a national average [43]. The estimated dietary intake from pasteurized full fat grade milk (3%) with a background level of 0.49 pg WHO-TEQ/g fat, would be 3.94 pg TEQ/day. Considering a 65 kg adult, the DL-PCBs estimated dietary intake would be 0.06 pg TEQ/kg of body weight/day. Since the calculated DL-PCBs estimated dietary intake is much smaller than the amounts declared by the World Health Organization as tolerable daily intake (1–4 pg WHOPCDD/F-PCB-TEQ/kg of body weight/day) the consumption of milk which is commercially available in local supermarket is not a threat for Iranian consumers [44].

## Conclusion

The present study showed that DL-PCBs and NDL-PCBs background levels in cow’s milk issued from different brands commercially available in Iran are similar to the levels found in EU countries. The levels of DL-PCBs and NDL-PCBs in all the analyzed samples were far below than the EU regulation for milk and the estimated intake levels of DL-PCBs were considered within the safe margin. However, due to existence of many known and unknown sources for PCBs contamination, large-scale production in the past and their bio-accumulative properties which may cause considerable environmental pollution, continuous monitoring of PCBs in environment especially in food is necessary. This residual characterization is also useful in HACCP system (hazard analysis critical control point) for ensuring the safety of food of animal source.

## Abbreviations

DL-PCBs: dioxin-like PCBs
HACCP: Hazard analysis critical control point
I.S.: Internal standard
LODs: Limits of detection LODs
LOQ: Limit of quantification
NDL-PCBs: Nondioxin-like PCBs
PCBs: Polychlorinated biphenyls
TEQ: Toxic equivalents
WHO: World Health Organization
WHO-TEF: WHO-toxic equivalency factors
